# Combating Two Major Fungal Diseases in Marigold (*Tagetes* spp.): Insights into Pathogen Identification and Antifungal Strategies

**DOI:** 10.3390/pathogens15090959

**Published:** 2026-09-09

**Authors:** Yi-Ping Zhang, Niaz Ali, Yue-Fei Li, Ci-Lin Yang, Wang-Qi Huang, Feng Xu, Xiu-Mei Yang, Shao-Fang Qian, Zheng-Song Lei, Ji-Hua Wang, Dong-Sheng Tang

**Affiliations:** 1 National Engineering Research Center for Ornamental Horticulture, Key Laboratory for Flower Breeding of Yunnan Province, Flower Research Institute, Yunnan Academy of Agricultural Sciences, Kunming 650200, Chinaniazaliadil@yahoo.com (N.A.);; 2 Department of Agriculture, Shaheed Benazir Bhutto University, Sheringal 18150, Pakistan; 3 International Agriculture Research Institute, Yunnan Academy of Agricultural Sciences, Kunming 650200, China; 4 State Key Laboratory for Conservation and Utilization of Bioresources in Yunnan, College of Plant Protection, Yunnan Agricultural University, Kunming 650201, China; 5 Yunnan Bohao Biotechnology Group Co., Ltd., Qujing 655331, China

**Keywords:** *Tagetes*, leaf spot, stem wilt, pathogen identification, *Alternaria alternata*, *Fusarium solani* species complex (FSSC)

## Abstract

Marigold (*Tagetes* spp.), a widely cultivated popular ornamental plant that is highly susceptible to fungal diseases, particularly leaf spot and stem wilt, which can substantially reduce its ornamental and economic value. This study aimed to identify the causal pathogens responsible for these diseases and evaluate the efficacy of selected antifungal agents against them. The causal agent of leaf spot was identified as *Alternaria alternata*, whereas stem wilt was caused by the *Fusarium solani* species complex (FSSC), based on Koch’s postulates, morphological characterization, and molecular identification. The antifungal activities of five commercially available fungicides were subsequently evaluated against both pathogens. The results showed that isomycete fluridine suspension (40%), metalaxyl–oxametrin suspension (32%), eugenol liquid (0.3%), pyrazolesterin methyl thiobacillin suspension (41%), and pyrazolesterin tebuconazole suspension (30%) demonstrated varying degrees of inhibitory activity. The *EC*50 values for *A. alternata* ranged from 10.588 to 49.604 mg/L, while those against FSSC ranged from 12.273 to 27.921 mg/L. These findings provide insights into the potency of these fungicides and offer a scientific basis for developing integrated disease management strategies for marigold cultivation, although further research is required to validate and evaluate their long-term efficacy and environmental safety.

## 1. Introduction

Marigold (*Tagetes erecta* L.) is an annual ornamental species belonging to the family Asteraceae and is cultivated under diverse agroclimatic conditions, owing to its ornamental, industrial, and medicinal value [1]. It is extensively grown for its ornamental blooms, natural pigments, essential oils, and bioactive compounds with therapeutic potential [2]. In ornamental horticulture, marigolds are planted in beds, borders and pots. The flower petals are a major source of xanthophyll pigments, particularly lutein, which constitute approximately 80–90% of the total carotenoid content [3,4,5]. Lutein extracted from marigold flowers is widely utilized in the poultry industry to enhance the pigmentation of egg yolks and broiler skin. Additionally, marigold-derived pigments have important applications in the textile, pharmaceutical, and nutraceutical industries [6]. Native to Mexico and Central America, marigolds were introduced to China in the 1990s due to their strong resistance, wide adaptability, and tolerance to both high and low temperatures, as well as wet and drought conditions. The crop is currently cultivated extensively throughout China at elevations ranging from approximately 1150 to 1680 m above sea level [7].

The cultivation area of pigment-producing marigold has expanded considerably in recent years. However, fungal diseases have become one of the major constraints limiting sustainable production. Among these diseases, leaf spot is particularly devastating because it significantly reduces flower yield and quality. *Alternaria zinnia* causes leaf spot and flower blight in marigold and may reduce crop yield by as much as 50–60% [8]. The disease has been reported in several countries, including Mexico, the United States, South Korea [9], India, Japan [10], and other regions [11,12]. Marigold leaf spot is also seed-borne, allowing fungal spores to survive within infected seeds and initiate disease development when favorable temperature and humidity conditions occur.

The causal organisms responsible for marigold leaf spot have been investigated extensively, although reports vary among geographical regions. The disease was first attributed to *Alternaria zinniae* in the United States during the 1940s [13]. Subsequent studies by Shome and Mustafee [14] and Mamata and Singh [15] identified *Alternaria tagetica* as the principal causal agent capable of infecting the entire plant. Similar findings have been reported from Atlanta, USA [16], Northwest India [17], Japan [10], and Taiwan, where *A. tagetica* was shown to infect leaves, stems, twigs, and flowers [18,19]. In China, however, *Alternaria alternata* was first reported as the causal agent of marigold leaf spot in Beijing [20]. More recently, *Alternaria cosmosa* was identified as the causal agent of marigold leaf blight in South Korea [21]. In addition to diseases caused by *Alternaria* species, marigold production is also affected by other important diseases, including white rot [22] and bacterial wilt [23].

While leaf spot has received considerable attention, stem wilt has recently emerged as another economically important disease affecting marigold production. *Fusarium solani,* a soil-borne fungal pathogen belonging to the *Fusarium solani* species complex (FSSC), causes severe diseases in a wide range of economically important crops [24]. Its host range includes legumes, cucurbits, potato, sweet potato, and several ornamental plant species. The pathogen is responsible for crown rot, stem rot, root rot, foot rot, damping-off, and vascular wilt, resulting in symptoms such as chlorosis, stunted growth, and, in severe cases, plant death [24,25]. For instance, *F. solani* f. sp. *eumartii* causes wilt and dry rot on potato (*Solanum tuberosum*) as well as foot rot on tomato (*Solanum lycopersicum*) [26]. In soybean (Glycine max), *F. solani* has been reported to cause severe root rot, significantly compromising plant stand establishment and reducing grain yield under field conditions [27]. Similarly, *F. solani* f. sp. *pisi* is responsible for root rot in garden pea (*Pisum sativum*), causing disease incidence of up to 32.43% and significant losses in pod and seed yield [28]. In the common bean, *F. solani* has also been recognized as one of the predominant root rot pathogens, with disease severity being negatively correlated with pod number and seed production [29].

To elucidate the major diseases affecting marigold production in Yunnan Province, China, we conducted a comprehensive field survey across the main marigold-producing regions. The survey revealed that leaf spot and stem wilt were the predominant diseases, characterized by high incidence rates and rapid disease progression. Without timely management, the disease can substantially reduce flower production and crop quality. Therefore, symptomatic plant samples were collected for pathogen isolation, morphological characterization, molecular identification, and pathogenicity verification. Notably, marigold stem wilt had not been previously reported in the scientific literature. In this study, FSSC was identified as the causal agent of marigold stem wilt.

Following pathogen identification, five commercially available fungicides were evaluated under laboratory conditions to determine their efficacy against the two major fungal pathogens. The objective of this study was to identify effective fungicides and their optimal concentrations for disease management while providing a scientific foundation for future integrated disease management strategies. The findings are expected to contribute to improved disease control, reduced yield losses, and more sustainable marigold production in Yunnan Province and other marigold-growing regions.

## 2. Materials and Methods

### 2.1. Investigation of the Incidence Rate of Leaf Spot and Wilt Pathogens in Marigold

#### 2.1.1. Investigation Site and Duration

The field survey was conducted from April to September 2022 in Lingjiao Village, Lingjiao Township, Zhanyi District, Qujing City, Yunnan Province (approximately 25.83° N, 103.88° E), a representative marigold (*Tagetes* spp.) cultivation region characterized by a subtropical monsoon climate. The multi-year average temperature is approximately 13.8 °C, with a large diurnal temperature variation and cool mornings and evenings. The experimental period (April–August) was characterized by concentrated rainfall, moderate temperatures, and variable weather conditions.

The investigation sites were part of the demonstration base of Yunnan Bohao Biotechnology Group Co., Ltd. (Kunming, China). All standard cultivation practices, including fertilization and chemical control, were implemented according to the company’s prescribed management protocols.

#### 2.1.2. Investigation Methods

A Z-shaped sampling pattern was employed based on the disease rating scales for marigold leaf spot and wilt diseases (Table 1 and Table 2). The number of diseased plants and the severity of symptoms were recorded. A total of 100 plants were surveyed. Field photographs of symptomatic plants were taken, and specimens exhibiting typical disease symptoms were collected and transported to the laboratory for microscopic examination and pathogen isolation. The disease incidence and disease index were calculated using the following formulas:

$$Disease\;incidence\;(\%)\;=\;\left.\left(\;\frac{Number\;of\;infected\;plants}{Total\;number\;of\;plants\;assessed}\right.\right)\times \;100$$

$$Disease\;index=\frac{\sum (Plants\;in\;grade\;I)\times (Grade\;value\;I)\;}{\left(Total\;plants\;surveyed)\times (Maximum\;grade\;value\right)}\times 100$$

#### 2.1.3. Experimental Materials

Plant samples of Marigold (*Tagetes* spp.) cv. ‘Bohao-1-hao’ exhibiting typical disease symptoms were collected from the cultivation facilities of Yunnan Bohao Biotechnology Group Co., Ltd., Yunnan, China. A total of twenty symptomatic tissue samples were collected, comprising ten leaf and ten stem samples. All samples were collected at the early stage of disease development to ensure optimal pathogen viability and representativeness of initial infection dynamics.

### 2.2. Isolation and Purification of Pathogenic Fungi

The pathogenic fungi were isolated and purified using the tissue separation method. Symptomatic marigold tissues were excised and thoroughly rinsed under running tap water to remove surface debris. The samples were surface-sterilized with 75% (*v*/*v*) ethanol for 30–60 s, followed by disinfection in 1.5% (*w*/*v*) sodium hypochlorite solution for 3–5 min. Subsequently, the tissues were rinsed three times with sterile distilled water and blotted dry on sterile filter paper to remove excess moisture.

Leaf and stem tissue sections (5 mm × 5 mm) were cut from the diseased–healthy junction of symptomatic plants, transferred to Petri plates containing potato dextrose agar (PDA), with four tissue pieces placed on each plate, and incubated at 25 °C for 3–4 days to allow fungal growth.

For single-spore purification, 5 mm × 5 mm pieces of fungal colonies were excised and subcultured on fresh PDA medium as described previously [30,31]. Subculturing was repeated three to five times until consistent colony morphology was achieved, indicating the successful establishment of pure fungal cultures.

### 2.3. Koch’s Postulates: Pathogenicity Test

To fulfill Koch’s postulates and conclusively establish the causal agent of the observed disease, pathogenicity assays were conducted using both in vitro and in vivo inoculation approaches.

#### 2.3.1. In Vitro Pathogenicity Assay

To verify the pathogenicity of the fungal isolate associated with marigold leaf spot, Koch’s postulates were fulfilled using an in vitro detached-leaf assay. The conidial suspension was prepared by harvesting spores from pure cultures of *Alternaria* and *Fusarium* isolates, suspending them in sterile distilled water, filtering the suspension through a 150-mesh sieve to remove mycelial debris, and adjusting the spore concentration to 1 × 10^6^ conidia/mL using a hemocytometer.

Healthy marigold leaves were thoroughly washed under running tap water, surface-sterilized with 70% ethanol, rinsed three times with sterile distilled water, and placed on sterile water agar (WA) in Petri dishes to maintain leaf turgidity during incubation. The leaf surfaces were gently wounded using the puncture inoculation method before inoculation with the conidial suspension [32]. Leaves treated with sterile distilled water served as the negative control (CK). Each treatment comprised three biological replicates.

Diseased symptom development was monitored daily. Characteristic leaf spot symptoms appeared 7–10 days post-inoculation (dpi). To complete Koch’s postulates, the pathogen was re-isolated from the symptomatic tissues using the tissue isolation method described above, and the recovered isolate was compared with the original culture based on morphological characteristics.

#### 2.3.2. In Vivo Pathogenicity Assay

The pathogenicity of the fungal isolate associated with marigold stem wilt was evaluated under in vivo conditions following Koch’s postulates. A spore suspension (1 × 10^6^ conidia/mL) prepared from purified cultures was used as the inoculum. The base of a healthy marigold stem was surface-sterilized with 75% ethanol, rinsed with sterile water and inoculated with the fungal spore suspension. Plants inoculated with sterile distilled water served as the negative control (CK).

Each treatment was performed in three biological replicates. The inoculated plants were maintained at room temperature (25 ± 1 °C), and disease symptom development was monitored daily. Upon typical stem wilt symptom appearance, the pathogen was re-isolated from infected stem tissues using the tissue separation method described previously [31,32]. The morphological characteristics of the re-isolated fungus were compared with those of the original isolate to confirm fulfillment of Koch’s postulates.

### 2.4. Identification of Pathogenic Fungi

#### 2.4.1. Morphological Identification

The purified representative strain was inoculated onto a PDA plate and incubated at 25 ± 1 °C in darkness with 60% relative humidity for 4 days. Colony morphology and medium color were directly observed, and the morphology, size, separation and arrangement of conidia were examined using a light microscope [31].

#### 2.4.2. Molecular Identification

Approximately 50 mg of fresh mycelium grown on a PDA solid medium was collected for genomic DNA extraction. Total genomic DNA was extracted using the MiniBEST Universal Genomic DNA Extraction Kit (Cat. No. 9765; Takara Bio Company, Kusatsu, Shiga, Japan) according to the manufacturer’s instructions and stored at −20 °C for subsequent use. For molecular identification, the internal transcribed spacer (ITS) region and the translation elongation factor 1-alpha (TEF1-α) gene were amplified using the following primer pairs:

- ITS region: ITS1 (5′-TCCGTAGGTGAACCTGCGG-3′) and ITS4 (5′-TCCTCCGCTTATTGATATGC-3′)
- TEF1-α gene: EF1 (5′-ATGGGTAAGGARGACAAGAC-3′) and EF2 (5′-GGARGTACCAGTSATCATG-3′) [33].

The PCR reaction mixture (25 µL) contained 2 µL of genomic DNA, 0.5 µL of each primer (10 µmol/L), 2.5 µL of 10× buffer, 2.5 µL of Mg^2+^ (25 mmol/L), 2.5 µL of dNTPs (10 µmol/L each), 0.5 µL of *Taq* DNA polymerase (5 U/µL), and 14 µL of ddH_2_O. The PCR amplification conditions were as follows: initial denaturation at 94 °C for 4 min; 35 cycles of denaturation at 94 °C for 30 s, annealing at 56 °C for 50 s, and extension at 72 °C for 1 min; and a final extension at 72 °C for 10 min. The products were held at 4 °C.

PCR amplicons (5 µL) were electrophoresed on a 1% agarose gel, visualized, and photographed. The PCR products were excised and sequenced by Fengke Biological Engineering Co., Ltd. (Shanghai, China). The obtained nucleotide sequences were compared with reference sequences available in the NCBI database using BLAST (≥90% sequence identity and query coverage, https://blast.ncbi.nlm.nih.gov, accessed on 29 June 2026) for preliminary species identification. Multiple sequence alignment was performed using MEGA 12 software. The best-fit nucleotide substitution model (GTR + G + I) was determined using the Akaike Information Criterion (AIC), and a multigene phylogenetic tree was subsequently constructed using the Maximum Likelihood (ML) method without an outgroup. Branch stability was evaluated through 1000 bootstrap replicates, providing robust resolution of the phylogenetic placement and confirming the species-level identity of the isolated pathogens.

### 2.5. In Vitro Screening of Fungicide for Disease Management

To identify effective fungicides against the isolated pathogens, five commercial fungicides were selected for in vitro evaluation (Table 3).

The antifungal activities of the selected fungicides were evaluated using the mycelial growth rate method as previously described [31]. Each fungicide was tested at five concentrations, with three biological replicates per concentration.

Stock solutions (1 × 10^5^ mg/L) were prepared for each fungicide and subsequently diluted to obtain tenfold concentrated working solutions. These solutions were then incorporated into sterilized potato dextrose agar (PDA) to achieve the desired final concentrations.

The concentrations evaluated were as follows: 32% metalaxyl-M·hymexazol at 25, 50, 100, 200, and 400 mg/L; 0.3% eugenol at 1, 2, 4, 8, and 16 mg/L; 41% pyraclostrobin·thiophanate-methyl at 0.5, 1, 2, 4, and 8 mg/L; and both 30% pyraclostrobin·tebuconazole and 40% iprodione·fluazinam at 0.1, 0.2, 0.4, 0.8, and 1.6 mg/L.

Sterilized PDA medium was cooled to 40–50 °C before the addition of the corresponding tenfold concentrated solution. The medium was mixed thoroughly to ensure uniform distribution of the fungicide and then poured into sterile Petri dishes. Three replicate plates were prepared for each concentration, while fungicide-free PDA plates served as untreated controls.

The pathogenic fungi were first cultured on PDA for 5 days to obtain actively growing colonies. A 5-mm-diameter mycelial plug was excised from the actively growing margin of each colony using a sterile hole punch and placed at the center of each fungicide-amended PDA plate. All inoculated plates were incubated at 25 ± 1 °C for 8 days. Colony diameter was measured using the cross-diameter method, and the percentage inhibition of mycelial growth was calculated using the following equation:

$$Inhibition\;Rate\;(\%)=\frac{C-T}{C}\times 100$$

where: C represents the colony diameter of the untreated control and T represents the colony diameter in the fungicide-treated culture.

Fungicides exhibiting higher inhibitory activity were selected for further sensitivity analysis. For each selected fungicide, five concentrations with approximately twofold serial dilutions were prepared as described above. A 5-mm-diameter mycelial plug from the actively growing margin of each fungal colony was placed at the center of a fungicide-amended PDA plate and incubated at 25 ± 1 °C for 7 days. Colony diameters were measured, and the inhibition rate was calculated relative to the untreated control. Dose–response curves were fitted using a four-parameter logistic (4-PL) nonlinear regression model to estimate the half-maximal effective concentration (EC_50_) and its 95% confidence interval (95% CI). The model was defined as:

$$Y=c+\frac{d-c}{1+{\left(\frac{X}{b}\right)}^{a}}$$

where ***Y*** is the relative inhibition rate, *X* is the fungicide concentration, *a* is the Hill slope, *b* is the EC_50_, and *c* and *d* represent the lower and upper asymptotes, respectively. Model parameters were estimated by nonlinear least-squares regression, and the goodness-of-fit was evaluated using the coefficient of determination (R^2^) [34,35].

### 2.6. Data Processing and Statistical Analysis

All experiments were conducted using a completely randomized design with three biological replicates. Statistical analyses were performed using SPSS version 19.0 (IBM Corp., Armonk, NY, USA). Data are presented as the mean ± standard deviation (SD). Differences among treatments were evaluated using a one-way analysis of variance (ANOVA), followed by Duncan’s multiple range test for mean separation. Differences were considered statistically significant at *p* < 0.05.

## 3. Results

### 3.1. Disease Incidence in Marigold Plantings in Qujing

A field survey was conducted to investigate the prevalence and severity of major diseases affecting marigold (*Tagetes erecta*) plantings under commercial cultivation conditions in Qujing, Yunnan Province. Two major diseases, leaf spot and *Fusarium* wilt, were consistently observed throughout the growing season. Diseases symptoms first appeared during the budding stage and progressively intensified, reaching their highest severity during the late flowering stage.

To quantitatively assess disease progression, leaf spot severity was classified using a 0–5 grading scale based on the percentage of foliar lesions (Table 1), while root and stem rot severity was evaluated according to the corresponding classification standard for vascular wilt symptoms (Table 2).

Using these criteria, the disease index (DI) for each growth stage was calculated. During the budding stage, leaf spot and Fusarium wilt remained relatively low, with average DIs of 8.3 and 5.7, respectively, corresponding to Grade 1 infections (Table 1 and Table 2). By the full-bloom stage, both diseases had intensified markedly. Leaf spot reached a moderate Grade 3 (DI = 42.6), while Fusarium wilt escalated to Grade 3 (DI = 38.1) as vascular discoloration became evident. The highest disease pressure was recorded during the late flowering stage, where the leaf spot severity peaked at Grade 4 (DI = 71.2), with coalescing necrotic lesions covering more than 50% of the leaf surface (Table 1). Concurrently, Fusarium wilt reached its maximum incidence at Grade 4 (DI = 68.5), characterized by severe wilting, stem cracking, and internal vascular browning (Table 2). These findings demonstrate that both foliar and vascular pathogens exhibit a synergistic progression, with disease severity closely correlating with the reproductive maturity of the host plant.

As shown in Table 4, the incidence of leaf spot increased markedly from 13.00% at the budding stage to 18.89% at the flowering stage and 71.67% at the late flowering stage. In contrast, the incidence of *Fusarium* wilt remained relatively low, increasing from 6.67% to 8%, and 10% at the respective developmental stages. Similarly, the disease index of leaf spot was consistently higher than that of *Fusarium* wilt across all growth stages, indicating that leaf spot was the predominant disease affecting marigold production in the study area (Table 4).

### 3.2. Pathogen Isolation and Verification of Pathogenicity

**Figure 1:**
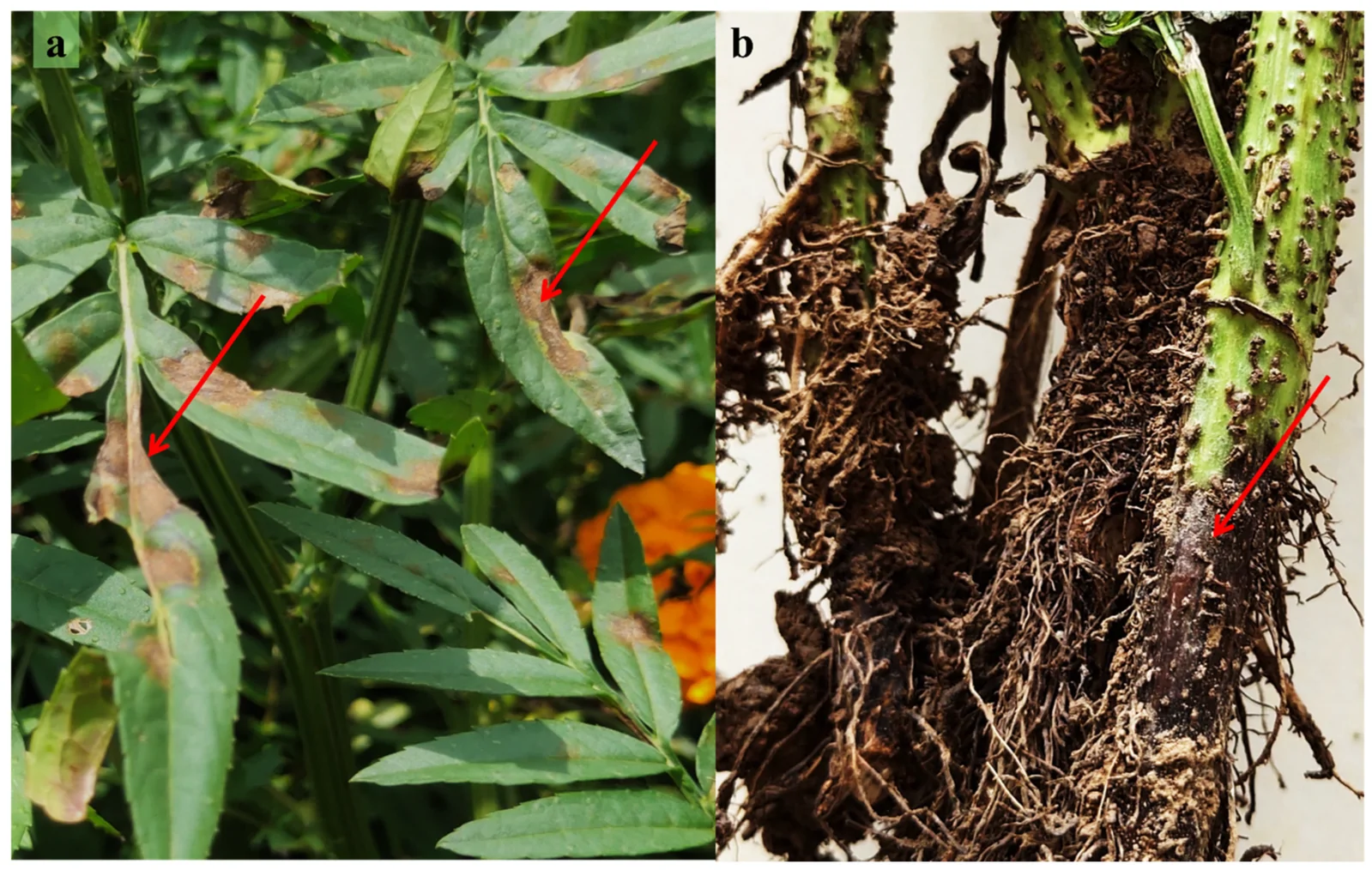
Disease symptoms in marigold (*Tagetes erecta*) at the flowering stage. (**a**) Foliar leaf spots. (**b**) Stem wilt caused by *Fusarium*. Arrows indicate lesion areas, highlighting the characteristic tissue degradation associated with progressive infection.

Typical symptoms of leaf spot initially appeared as small brown lesions on leaves, which gradually enlarged and extended to the stem, resulting in extensive tissue necrosis (Figure 1a). In wilted plants, dark brown discoloration developed at the stem base, and longitudinal sections revealed abundant floccose fungal mycelia within the vascular tissues (Figure 1b).

A total of ten symptomatic samples, comprising five leaf spot and five stem wilt samples, were processed for pathogen isolation. Following tissue isolation and single-spore purification, ten representative fungal isolates were obtained. Isolate recovered from leaf spot lesions were designated HBB1–HBB5, whereas those isolated from wilted stems were designated KWB1–KWB5.

**Figure 2:**
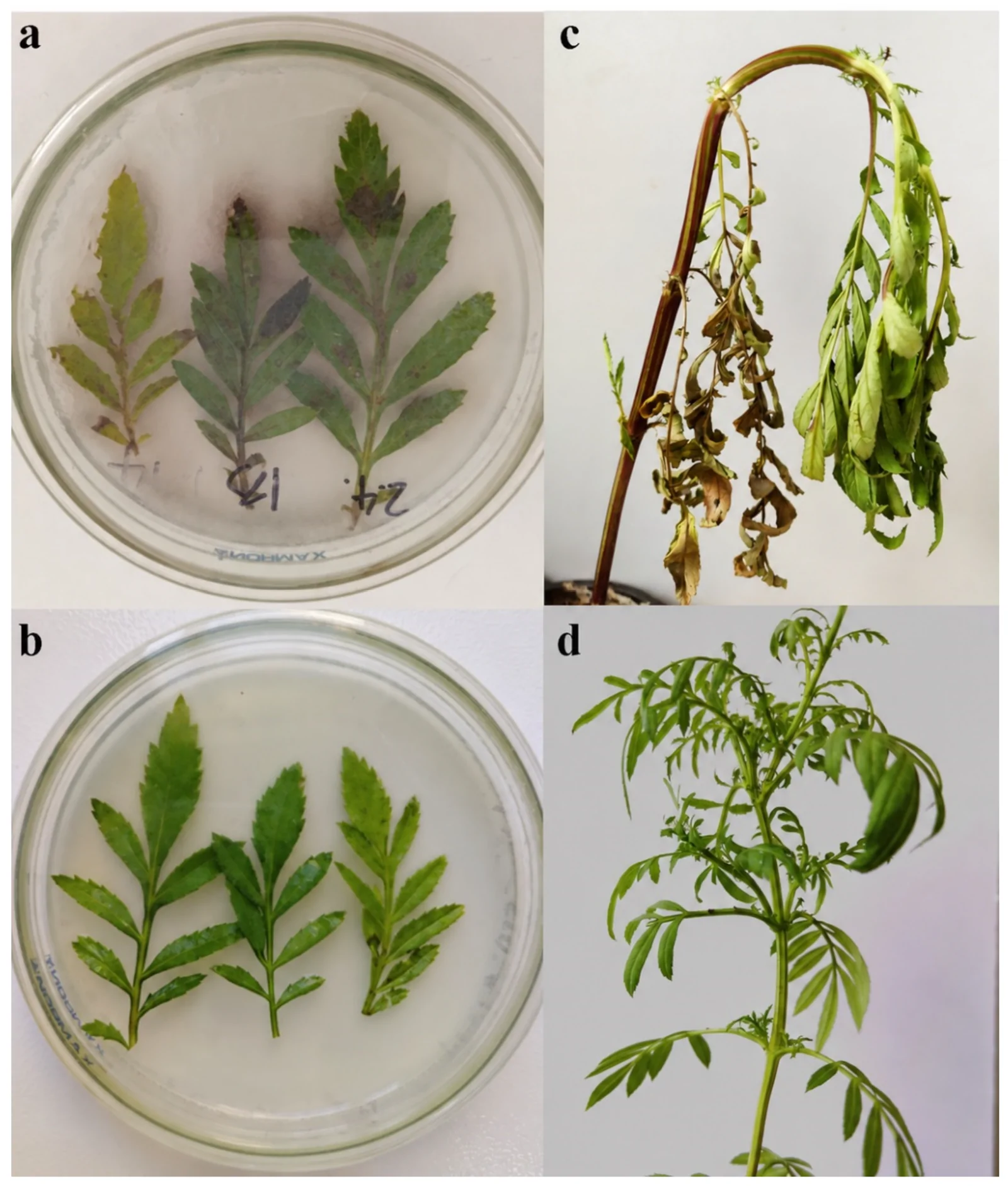
Pathogenicity assays of representative fungal isolates on marigold at flowering stage. (**a**) Detached leaves inoculated with HBB. (**b**) Non-inoculated control. (**c**) Whole plants inoculated with KWB. (**d**) Non-inoculated control.

The pathogenicity of representative isolates was confirmed by fulfilling Koch’s postulates. Following inoculation with the HBB isolate, visible browning developed around the wound site on detached petals by 4 dpi. By 6 dpi, the petal tips exhibited extensive chlorosis and necrosis, accompanied by abundant fungal colonization. Detached leaf assays showed initial chlorosis at 5 dpi, followed by the development of dark brown necrotic lesions that progressively expanded until the entire leaf became necrotic by 8 dpi, with profuse mycelial growth visible on the leaf surface (Figure 2a,b).

Plants inoculated with the representative KWB isolate developed typical wilt symptoms under greenhouse conditions. By 23 dpi, infected plants exhibited wilting, beginning with the basal leaves and subsequently spreading upward until severe plant collapse occurred (Figure 2c). The same fungal isolate was consistently re-isolated from symptomatic tissues, thereby satisfying Koch’s postulates and confirming that HBB and KWB isolates were the causative agents of marigold leaf spot and stem wilt, respectively.

### 3.3. Morphological Identification of Pathogenic Fungi

Colony morphology and microscopic characteristics of the representative HBB and KWB isolates were examined after culture on PDA.

**Figure 3:**
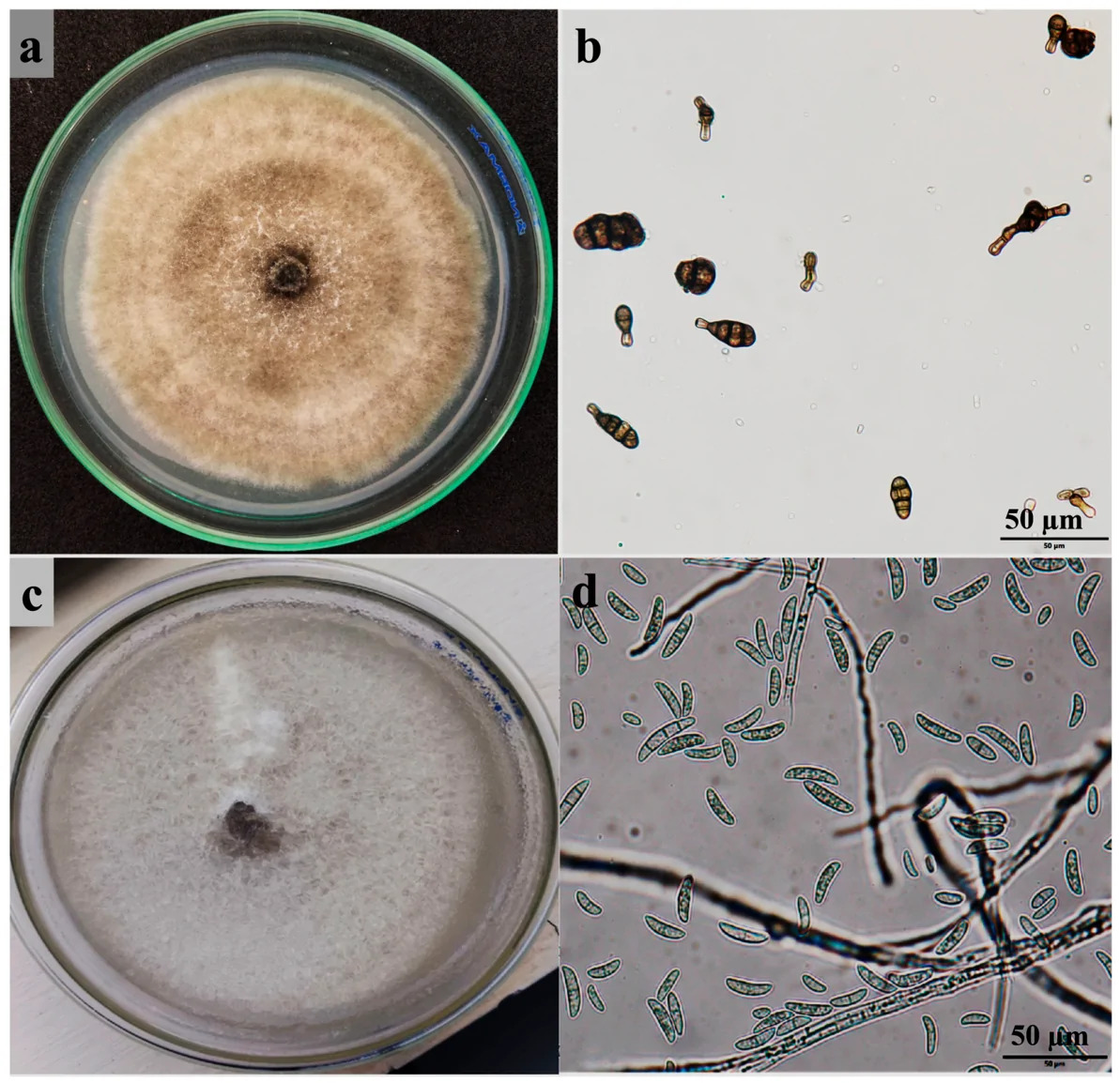
Morphological characterization of the fungal pathogens isolated from marigold (*Tagetes erecta*). (**a**) Colony morphology of *Alternaria alternata* isolate HBB on PDA. (**b**) Conidia of *A. alternata*. (**c**) Colony morphology of FSSC isolate KWB on PDA. (**d**) Microconidia of FSSC. Scale bars = 50 μm.

The HBB isolate produced rapidly expanding colonies with abundant floccose to cottony aerial mycelia. Colonies were initially grayish white and gradually became dark gray to olive-brown with age (Figure 3a). Microscopic examination revealed obclavate to ellipsoidal conidia with distinct transverse septa, characteristic of *Alternaria* species. Conidia measured 9.23–22.93 μm × 6.61–15.63 μm and generally possessed 1–3 transverse septa (Figure 3b).

In contrast, the KWB isolate formed dense, white floccose colonies during the early stages of growth, which gradually developed a characteristic pale to rose-red pigmentation upon maturity (Figure 3c). Only microconidia were observed. The conidia were hyaline, oval to ellipsoidal, predominantly aseptate, measuring 4.19–10.71 µm × 1.68–4.71 µm (Figure 3d). The observed morphological characteristics were consistent with those previously reported for *Fusarium solani*.

### 3.4. Molecular Identification of Pathogenic Fungi

To confirm the taxonomic identity of the representative isolates, genomic DNA was extracted from single-spore purified cultures for molecular characterization. The internal transcribed spacer (ITS) region was amplified and sequenced for both HBB and KWB isolates, while the translation elongation factor1-alpha (TEF-1α) gene was additionally amplified for the KWB isolate to improve species-level resolution.

**Figure 4:**
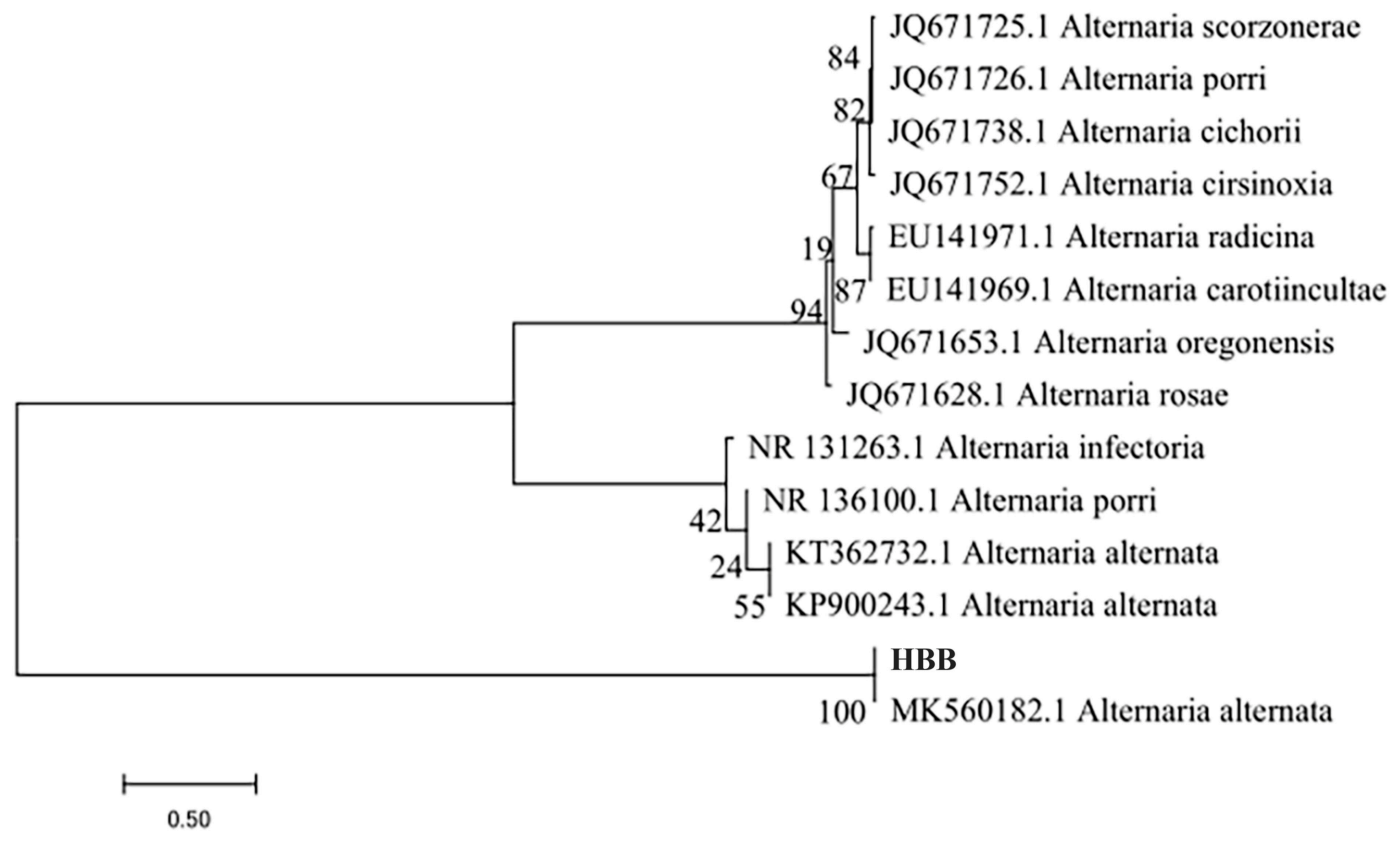
Phylogenetic tree constructed based on combined ITS and TEF gene sequences of the representative HBB isolate associated with marigold leaf spot.

Phylogenetic analysis based on ITS and TEF-1α sequences placed the cluster with the reference isolate of *Alternaria alternata* with 100% bootstrap support (Figure 4). The ITS sequence was deposited in GenBank under accession number ON489303.

**Figure 5:**
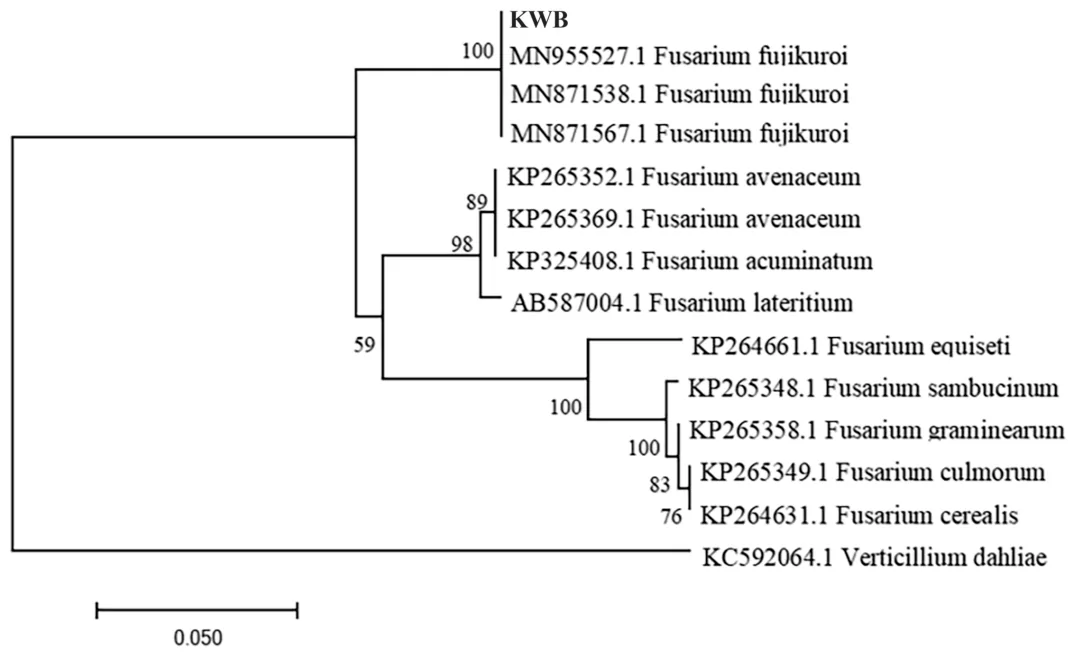
Phylogenetic tree constructed based on ITS and TEF gene sequences of the representative KWB isolate associated with marigold Fusarium wilt.

Similarly, a combined analysis of the ITS and TEF-1α sequences placed the KWB isolate within the *Fusarium solani* species complex, also with 100% bootstrap support (Figure 5). The ITS and TEF sequences were deposited in GenBank under accession numbers PZ092901 and PZ000720, respectively.

### 3.5. In Vitro Fungicide Sensitivity of Alternaria alternata

**Figure 6:**
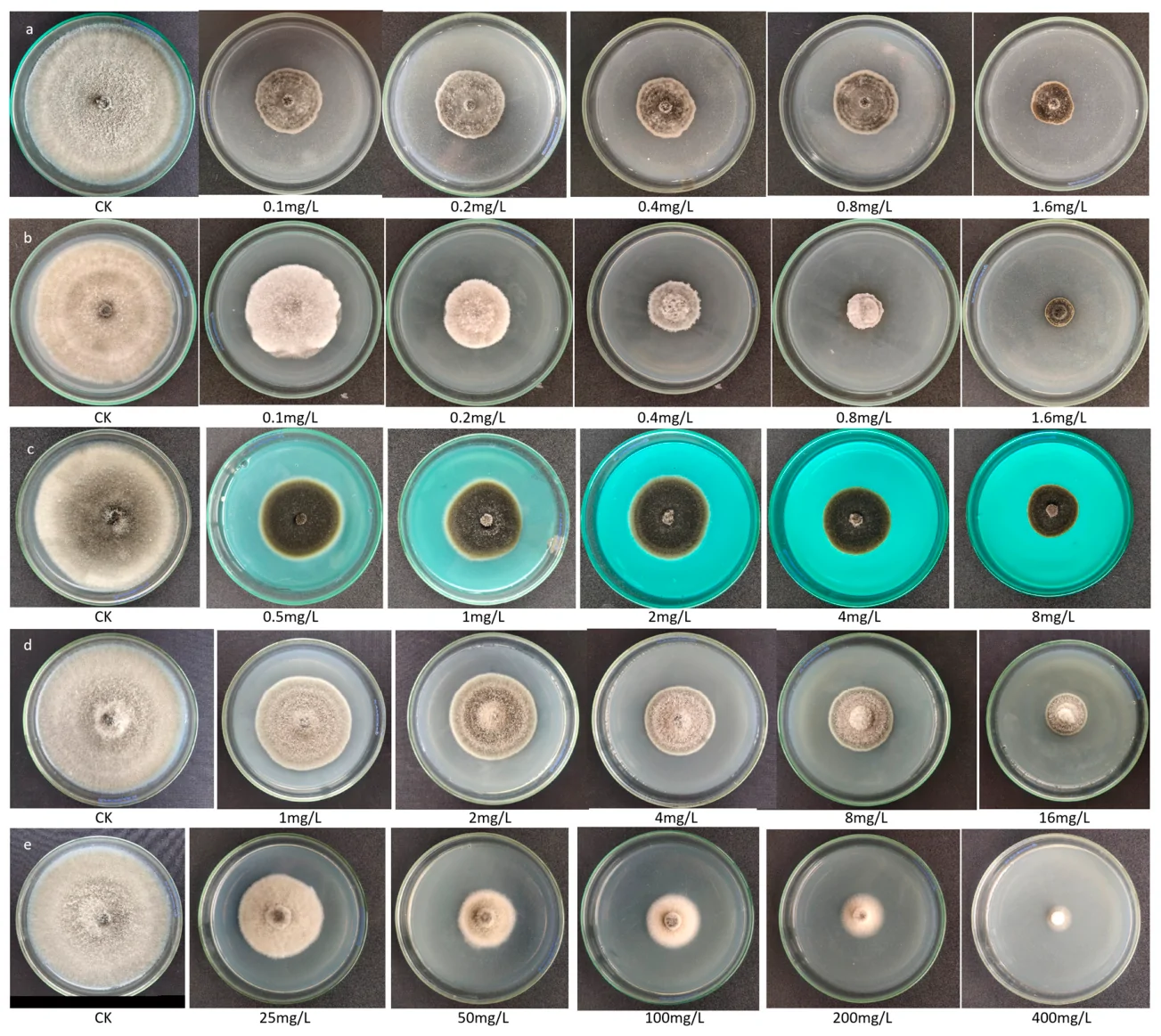
Inhibitory effect of five fungicides on mycelial growth of *Alternaria alternata*. (**a**) Pyraclostrobin–tebuconazole suspension; (**b**) iprodione–fluazinam suspension; (**c**) pyraclostrobin–thiophanate-methyl suspension seed coat agent; (**d**) eugenol solution; (**e**) metalaxyl-M–hymexazol suspension seed treatment liquid.

**Figure 7:**
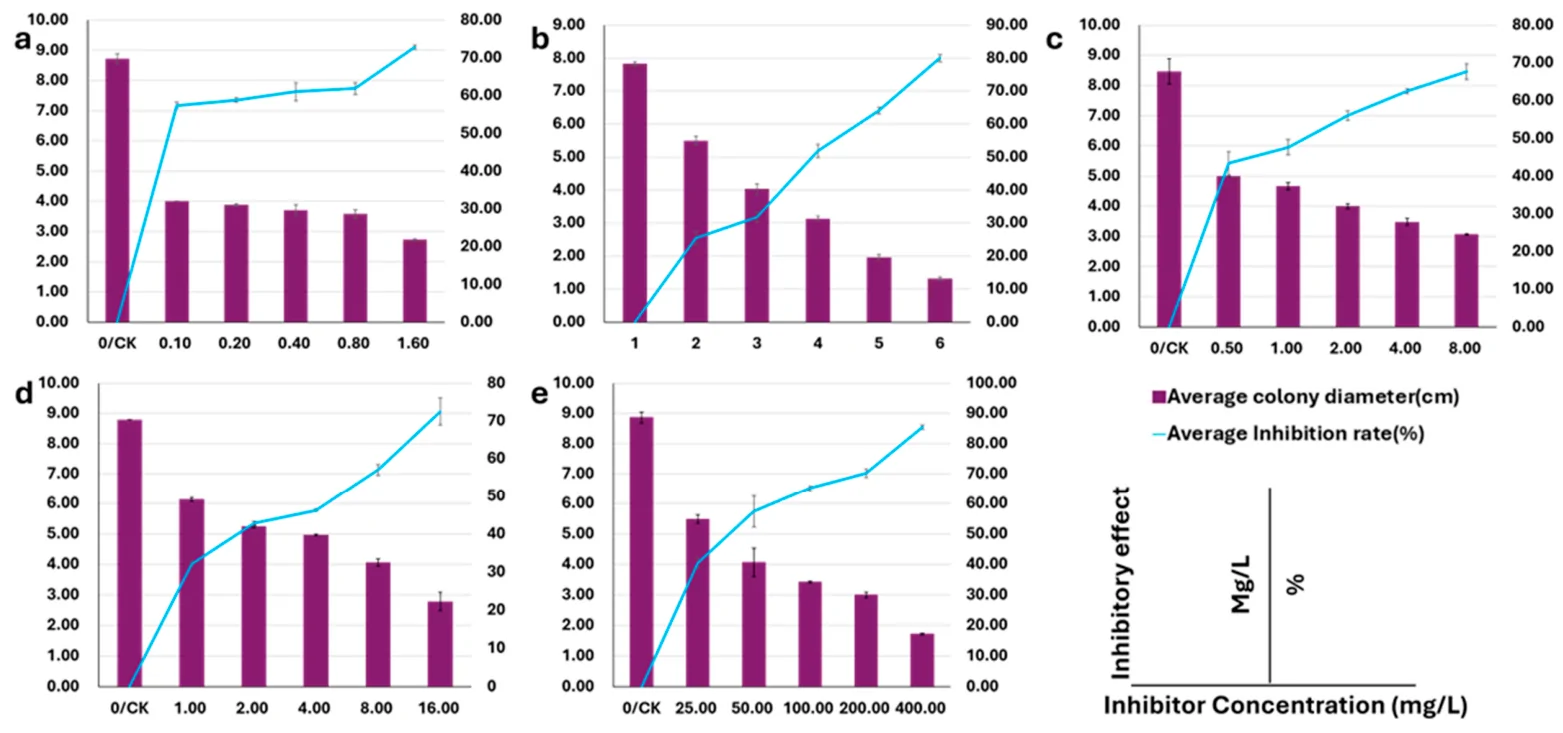
Inhibitory effects of fungicides on mycelial growth of *Alternaria alternata*. (**a**) Pyraclostrobin–thiophanate-methyl suspension seed-coating agent; (**b**) eugenol soluble agent; (**c**) metalaxyl-M–hymexazol seed-treatment liquid; (**d**) iprodione–fluazinam suspension; and (**e**) pyraclostrobin–tebuconazole suspension. Error bars indicate standard error (SE).

The inhibitory effects of five commercial fungicides against *Alternaria alternata* were evaluated using the mycelial growth inhibition assay. All tested fungicides significantly suppressed mycelial growth. However, their inhibitory capacities varied among treatments. (Figure 6 and Figure 7). Based on overall inhibition efficiency, the fungicides were ranked as follows: 40% iprodione–fluazinam suspension > 30% pyraclostrobin–tebuconazole suspension > 41% pyraclostrobin–thiophanate-methyl suspension seed coating agent > 32% metalaxyl-M–hymexazol suspension seed treatment liquid > 0.3% eugenol solution. As shown in Figure 7, 40% iprodione–fluazinam suspension exhibited inhibition rates ranging from 25.44% to 80.00% at concentrations of 0.1–1.6 mg/L. The inhibition rates of 30% pyraclostrobin–tebuconazole suspension ranged from 57.39% to 72.81% within the same concentration range. For 0.3% eugenol solution, inhibition increased from 32.13% to 72.49% at concentrations of 1–16 mg/L. Similarly, a 41% pyraclostrobin–thiophanate-methyl suspension seed-coating agent inhibited mycelial growth by 43.41–67.71% at concentrations of 0.5–8 mg/L, whereas a 32% metalaxyl-M–hymexazol suspension seed treatment liquid showed inhibition rates of 43.40–67.17% at concentrations of 25–400 mg/L.

At relatively low concentrations, the 30% pyraclostrobin–tebuconazole suspension exhibited stronger initial inhibition than the 40% iprodione–fluazinam suspension. However, when the concentration increased to ≥0.4 mg/L, iprodione–fluazinam showed superior inhibitory activity. Across the tested concentration gradients, the inhibition rate of pyraclostrobin–tebuconazole increased by 15.42% (from 57.39% to 72.81%), whereas iprodione–fluazinam demonstrated a substantially greater increase of 54.56% (from 25.44% to 80.00%), indicating a stronger concentration-dependent inhibitory response.

The fungicidal efficacy against A. alternata was further evaluated using EC_50_ values (Table 5). The fungicides were ranked according to increasing EC_50_ values as follows: 40% iprodione–fluazinam suspension (10.588 mg/L) > 32% metalaxyl-M–hymexazol suspension seed treatment liquid (16.961 mg/L) > 0.3% eugenol solution (17.518 mg/L) > 41% pyraclostrobin–thiophanate-methyl suspension seed-coating agent (26.479 mg/L) > 30% pyraclostrobin–tebuconazole suspension (49.604 mg/L). Lower EC_50_ values indicated stronger antifungal activity. The regression models for all fungicides, except the 30% pyraclostrobin–tebuconazole suspension, exhibited R^2^ values >0.90, demonstrating a strong correlation between fungicide concentration and inhibition response.

### 3.6. In Vitro Fungicide Sensitivity of FSSC

**Figure 8:**
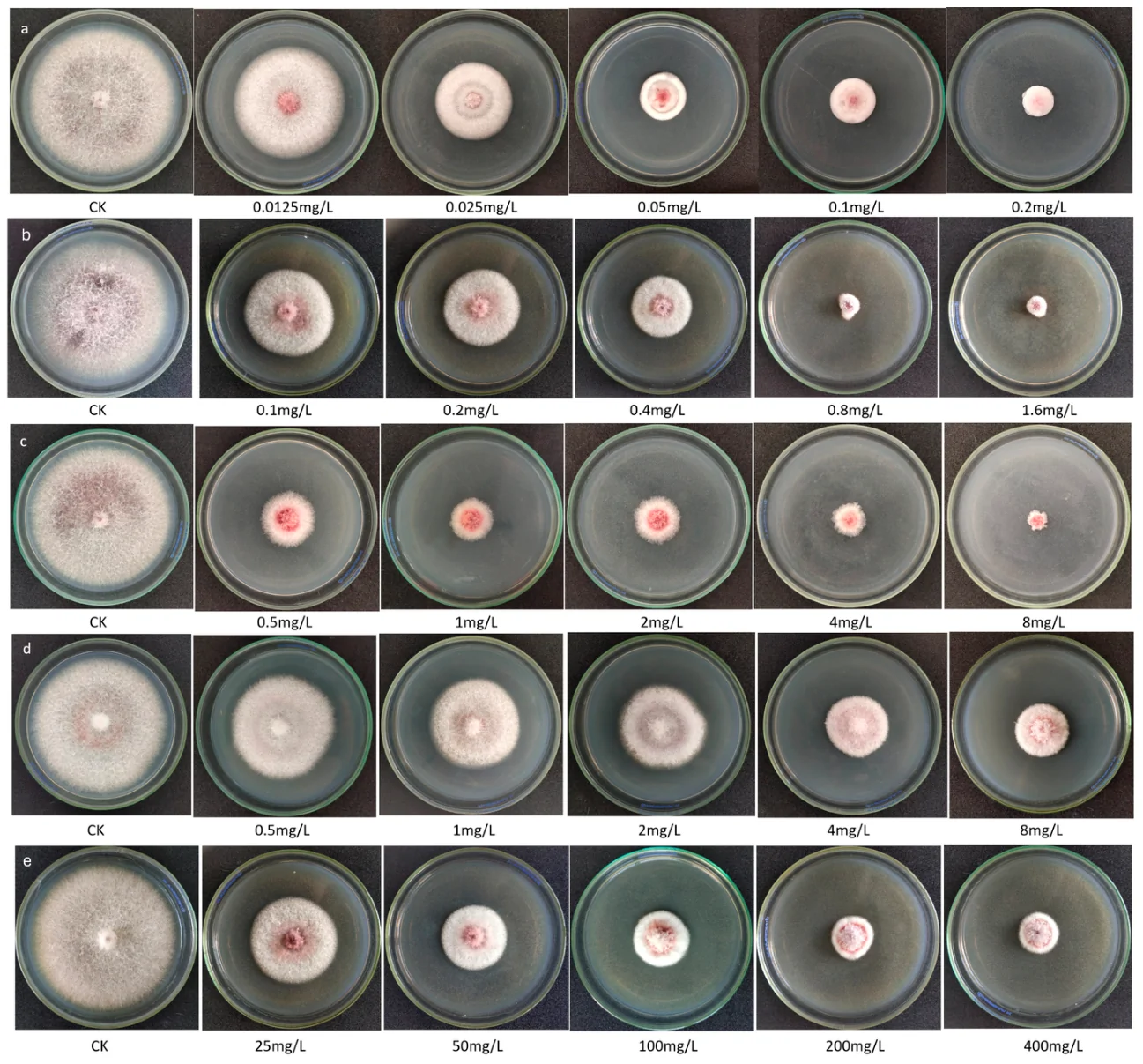
Inhibitory effects of five fungicides on mycelial growth of FSSC. (**a**) Iprodione–fluazinam suspension; (**b**) pyraclostrobin–thiophanate-methyl suspension seed-coating agent; (**c**) pyraclostrobin–tebuconazole suspension; (**d**) eugenol suspension; (**e**) metalaxyl-M–hymexazol suspension seed treatment liquid.

**Figure 9:**
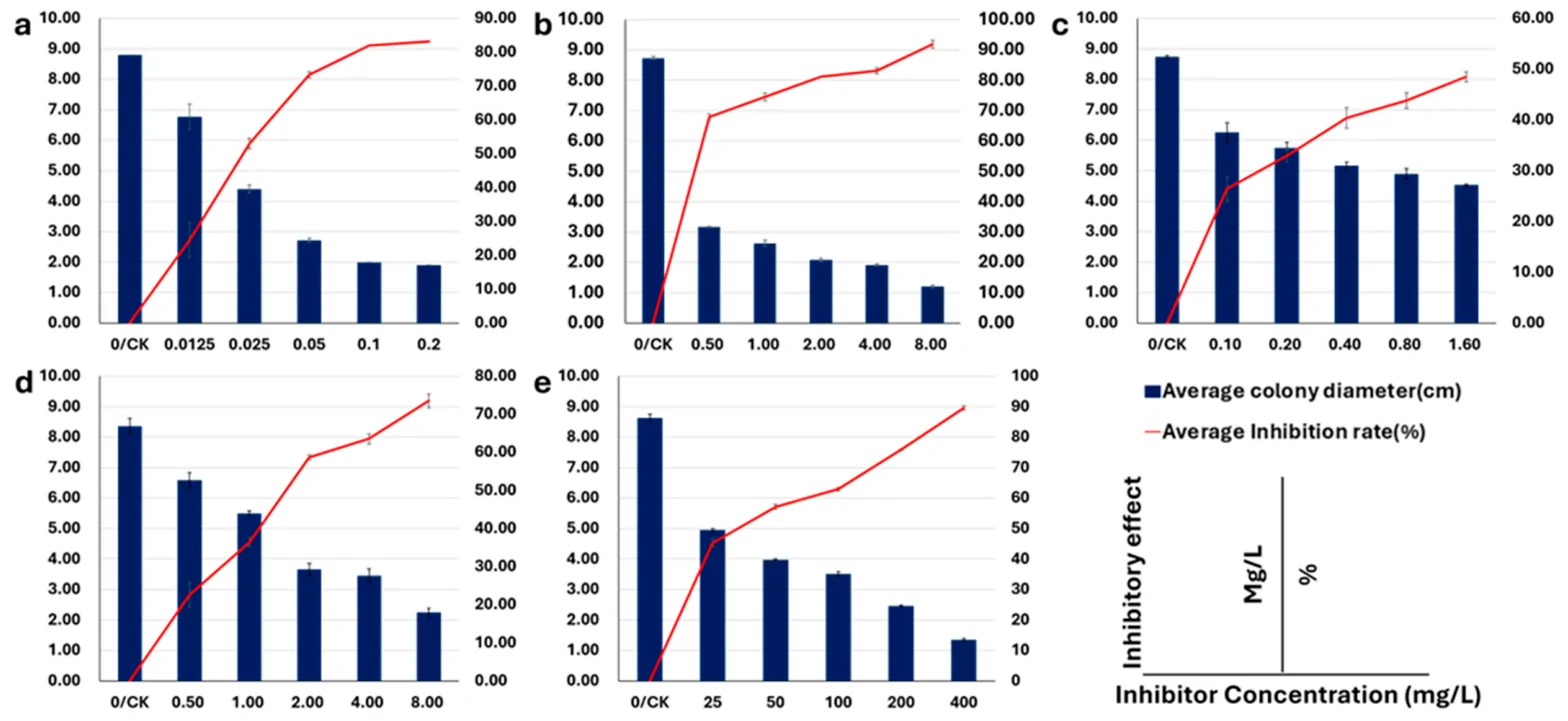
Inhibitory effects of fungicides on mycelial growth of Fusarium solani species complex (FSSC). (**a**) Iprodione–fluazinam suspension; (**b**) pyraclostrobin–thiophanate-methyl suspension seed-coating agent; (**c**) pyraclostrobin–tebuconazole suspension; (**d**) Eugenol soluble agent; and (**e**) metalaxyl-M–hymexazol seed-treatment liquid. Error bars indicate standard error (SE).

The inhibitory effects of the five fungicides against FSSC were also evaluated using the mycelial growth inhibition assay. All tested fungicides significantly inhibited fungal growth, although their effectiveness differed among treatments (Figure 8 and Figure 9). The overall inhibitory efficacy was ranked as follows: 40% iprodione–fluazinam suspension > 30% pyraclostrobin–tebuconazole suspension > 41% pyraclostrobin–thiophanate-methyl suspension seed-coating agent > 0.3% eugenol solution > 32% metalaxyl-M–hymexazol suspension seed treatment liquid.

According to Figure 9, the 30% pyraclostrobin–tebuconazole suspension exhibited inhibition rates ranging from 74.59% to 91.87% at concentrations of 1–8 mg/L. The inhibition rates of the 32% metalaxyl-M–hymexazol suspension seed treatment liquid ranged from 45.27% to 89.55% at concentrations of 25–400 mg/L. For the 40% iprodione–fluazinam suspension, inhibition increased from 24.50% to 83.13% at concentrations of 0.0125–0.2 mg/L. Meanwhile, the 41% pyraclostrobin–thiophanate-methyl suspension seed-coating agent exhibited relatively lower inhibition, ranging from 26.41% to 48.50% at concentrations of 0.1–1.6 mg/L.

Based on EC_50_ values, the antifungal efficacy of the tested fungicides against FSSC was ranked as follows (Table 5): 40% iprodione–fluazinam suspension (12.273 mg/L) > 0.3% eugenol solution (15.083 mg/L) > 32% metalaxyl-M–hymexazol suspension seed treatment liquid (15.579 mg/L) > 41% pyraclostrobin–thiophanate-methyl suspension seed-coating agent (27.124 mg/L) > 30% pyraclostrobi–·tebuconazole suspension (27.921 mg/L).

Among all tested fungicides, the 40% iprodione–fluazinam suspension exhibited the strongest inhibitory activity against both *A. alternata* and FSSC, suggesting its potential as an effective candidate for controlling major fungal diseases of marigold. The regression models showed strong goodness-of-fit, with R^2^ values exceeding 0.90 for all treatments except the 40% iprodione–fluazinam suspension, indicating a reliable relationship between fungicide concentration and mycelial growth inhibition.

## 4. Discussion

In the present study, two major fungal pathogens associated with marigold (*Tagetes erecta*) diseases were identified: *Alternaria alternata* as the causal agent of leaf spot disease and *Fusarium solani* species complex (FSSC) as the pathogen responsible for stem wilt. The identification results differ partially from those reported in previous studies, reflecting the diversity of the fungal pathogens associated with marigold diseases. Several Alternaria species, including *A. tagetica* [10,14,16,18,19], *A. zinniae* [36], *A. brassicae* [37], and *A. alternata* [20], have previously been reported as causal agents of marigold leaf spot. In the present study, the leaf spot pathogen affecting marigold in Yunnan Province was identified as *A. alternata* based on morphological characteristics and ITS sequence analysis, which is consistent with the findings by Li et al. [20], who identified *A. alternata* as the causal agent of marigold leaf spot in Beijing. These findings further expand the known distribution of *A. alternata*-associated leaf spot in marigold-growing regions.

Marigold leaf spot may be associated with seedborne inoculum. Therefore, seed treatment could represent a useful strategy for reducing early pathogen establishment and disease development. In this study, 32% metalaxyl-M–hymexazol seed treatment liquid and 41% pyraclostrobin–thiophanate-methyl suspension seed-coating agent exhibited moderate inhibitory effects against *A. alternata,* with inhibition rates of 67.17% and 67.71%, respectively. Additional seed treatment fungicides, including metalaxyl-M–fludioxonil, difenoconazole, and tebuconazole, could be evaluated in future studies to identify more effective control options. Previous research by Satapathy and Sahoo [8] reported that azoxystrobin + tebuconazole, tebuconazole + sulphur, and metalaxyl produced mycelial growth inhibition rates of 96.85%, 96.40%, and 95.50%, respectively, against *Alternaria zinniae*. Similarly, the present study demonstrated that pyraclostrobin-based formulations, particularly 41% pyraclostrobin–thiophanate-methyl and 30% pyraclostrobin–tebuconazole, exhibited substantial inhibitory activity against *A. alternata.* This antifungal activity is likely attributable to the mode of action of strobilurin fungicides, as pyraclostrobin and azoxystrobin inhibit mitochondrial respiration by binding to the cytochrome *bc1* complex, thereby disrupting fungal energy production.

Stem wilt caused by FFSC is a typical soil-borne disease, making early-stage management through seed treatment an important prevention strategy. In the present study, 40% iprodione–fluazinam suspension and 30% pyraclostrobin–tebuconazole suspension exhibited strong inhibitory effects against FSSC, with inhibition rates of 83.13% and 91.87%, respectively. Previous studies have shown that fludioxonil + metalaxyl-M and hymexazol effectively suppress *Fusarium oxysporum* f. sp. *melonis* in melon production systems [38]. Consistent with these findings, 32% metalaxyl-M–hymexazol seed treatment liquid also exhibited considerable inhibitory activity against FSSC in the present study. Collectively, these results suggest that fungicide mixtures containing multiple active ingredients may provide enhanced protection against soil-borne *Fusarium* diseases through complementary modes of action.

All five commercial fungicides significantly inhibited the mycelial growth of both *Alternaria alternata* and FSSC, although their antifungal efficacy varied considerably among treatments. These differences are likely attributable to the distinct modes of action of their active ingredients. Metalaxyl-M is a systemic phenylamide fungicide that effectively suppresses seed- and soil-borne pathogens by inhibiting RNA synthesis in oomycetes, while hymexazol is readily absorbed by plant roots and provides protection against soil-borne diseases through its fungitoxic activity and rhizosphere persistence. Eugenol, a naturally occurring phenolic compound derived from clove oil, exhibits broad-spectrum antifungal activity by disrupting membrane integrity, increasing cellular permeability, and interfering with mitotic processes [39]. Pyraclostrobin, a quinone outside inhibitor (QoI) fungicide, suppresses fungal development by inhibiting mitochondrial respiration and ATP production, thereby providing protective, curative, and translaminar activity [39]. Tebuconazole, a triazole fungicide, inhibits ergosterol biosynthesis by targeting sterol 14α-demethylase, resulting in impaired fungal cell membrane formation and growth [40]. Fluazinam acts as an uncoupler of oxidative phosphorylation, disrupting cellular energy metabolism [41], whereas iprodione interferes with fungal signal transduction and developmental processes, thereby inhibiting mycelial growth and sporulation [42]. Thiophanate-methyl, a benzimidazole fungicide, is converted to carbendazim in fungal cells, where it binds to β-tubulin and disrupts microtubule assembly, ultimately inhibiting mitosis and cell division [43,44]. The superior performance of fungicide mixtures observed in the present study may, therefore, be attributed to the complementary and potentially synergistic interactions among active ingredients with different biochemical targets. Such multi-site or multi-mechanistic activity not only broadens the spectrum of pathogen control but may also enhance disease suppression and contribute to delaying the evolution of fungicide resistance in pathogen populations.

Although the present study provides valuable insights into the antifungal efficacy of the tested fungicides under controlled in vitro conditions, these findings should be regarded as preliminary indicators of their potential performance. The effectiveness of fungicides under field conditions may differ substantially due to environmental and biological factors, including rainfall, temperature, ultraviolet radiation, plant physiology, pathogen–host interactions, soil characteristics, and microbial communities. Consequently, greenhouse and multi-location field trials are essential to validate the practical efficacy of these fungicides and to optimize application timing, dosage, and disease management strategies under commercial production conditions.

Beyond chemical control, long-term and sustainable control of marigold leaf spot and stem wilt requires an integrated disease management (IDM) approach that combines cultural, biological, and chemical measures. Exclusive reliance on fungicides may accelerate the development of resistant pathogen populations while raising environmental and ecological concerns. Therefore, integrating non-chemical approaches can improve disease suppression and reduce dependence on synthetic fungicides. Recommended cultural practices include maintaining appropriate planting density, improving field aeration, optimizing irrigation and fertilization, preventing prolonged soil moisture and waterlogging, implementing crop sanitation, and removing infected plant debris to minimize pathogen inoculum.

Biological control strategies also offer environmentally sustainable alternatives for disease management. Previous studies have demonstrated that marigold-derived phytochemicals possess inhibitory activity against important plant pathogens, including *Alternaria alternata* and *Fusarium oxysporum* [45]. Likewise, beneficial microorganisms such as *Trichoderma asperellum*, *T. harzianum*, *T. virens*, and *Bacillus* spp. have consistently exhibited antagonistic activity against *Alternaria* and *Fusarium* species through mechanisms including mycoparasitism, competition, and induction of host resistance [46,47]. In addition, plant-derived compounds such as *Eucalyptus globulus* essential oil have shown promising antifungal activity against *Alternaria* species [48], while *Beauveria bassiana* has demonstrated inhibitory effects against *Fusarium* pathogens [49]. Continued advances in pathogen biology, molecular diagnostics, population genetics, and genomics will further improve our understanding of pathogen diversity, epidemiology, and host–pathogen interactions, thereby facilitating the development of more precise and sustainable disease management strategies [50,51].

Overall, this study provides a comprehensive assessment of the major fungal pathogens associated with marigold leaf spot and stem wilt and identifies effective fungicide options for their management. The integration of accurate pathogen identification with comparative fungicide evaluation establishes a scientific foundation for evidence-based disease management. Future research should emphasize field validation of the most effective fungicides, optimization of fungicide rotation and mixture programs, resistance monitoring, and the integration of biological control agents with conventional management practices. Such integrated approaches will contribute to sustainable disease management, prolong fungicide efficacy, and enhance the productivity and health of marigold cultivation.

## 5. Conclusions

This study provides new insights into the identification and management of major fungal diseases affecting marigold (*Tagetes erecta*) production in Yunnan Province. Through a combination of morphological characterization, pathogenicity assays, and molecular identification, *Alternaria alternata* and FSSC were confirmed as the causal agents of leaf spot and stem wilt, respectively.

Field surveys demonstrated that leaf spot disease showed consistently higher incidence and disease severity compared with stem wilt across different growth stages, indicating that *A. alternata*-associated leaf spot represents a major threat to marigold cultivation. Fungicide sensitivity assays revealed significant differences in pathogen responses among the tested treatments. Among the five evaluated fungicides, the 40% iprodione·fluazinam suspension exhibited the strongest overall inhibitory activity against both pathogens, whereas the 30% pyraclostrobin·tebuconazole suspension showed particularly high efficacy against FSSC.

Collectively, these findings establish a scientific foundation for the integrated management of marigold fungal diseases by combining reliable pathogen identification with targeted fungicide selection. Although the fungicide evaluations were conducted under controlled in vitro conditions, the results provide valuable guidance for future disease management strategies. Further greenhouse and field investigations, together with the evaluation of biological control agents and sustainable management practices, are required to develop effective and environmentally compatible approaches for long-term marigold production.

## Figures and Tables

**Table 1:** Grading Standards for Marigold Leaf Spot Disease.

Disease Grade	Representative Value	Representative Symbol	Degree of Harm	Grading Standard
I	0	-	No harm	Healthy; no symptoms
II	1	+	Slight	Leaf lesion area ≤ 5%
III	2	++	Moderate	5% < leaf lesion area ≤ 15%
IV	3	+++	Severe	Leaf lesion area > 15%

Note: Leaf lesion area = percentage of total leaf surface affected. ‘-’ = no symptoms; ‘+’ = mild; ‘++’ = moderate; ‘+++’ = severe.

**Table 2:** Classification standard of root and stem diseases of *Tagetes erecta*.

Disease Grade	Representative Value	Representative Symbol	Degree of Harm	Grading Standard
I	0	-	No harm	Healthy; no symptoms
II	1	+	Slight	Stem lesion area ≤ 10% AND leaf/top leaf wilting ≤ 10%
III	2	++	Moderate	10% < stem lesion area ≤ 20% AND 10% < leaf/top leaf wilting ≤ 20%
IV	3	+++	Severe	Stem lesion area > 20% AND leaf/top leaf wilting > 20%

Note: Stem lesion area = percentage of total stem surface affected; leaf/top leaf wilting = percentage of foliage affected. ‘-’ = no symptoms; ‘+’ = mild; ‘++’ = moderate; ‘+++’ = severe.

**Table 3:** Commercial fungicides used in the in vitro antifungal assay against *Alternaria* and *Fusarium* (FSSC) isolates.

Code No.	Trade Name	Active Ingredient	Manufacturer
1	32% metalaxyl-M·hymexazol suspension seed treatment liquid	4% metalaxyl-M, 28% hymexazol	Jiangsu Mingde Lida Crop Technology Co., Ltd., Huaian, China
2	0.3% eugenol liquid	0.3% eugenol	Jiangsu Nantong Shenyu Green Pharmaceutical Co., Ltd., Nantong, China
3	30% pyraclostrobin·tebuconazole suspension	20% tebuconazole, 10% pyraclostrobin	Jiangsu Mingde Lida Crop Technology Co., Ltd., Huaian, China
4	40% iprodione·fluazinam suspension	20% iprodione, 20% fluazinam	Jiangsu Mingde Lida Crop Technology Co., Ltd., Huaian, China.
5	41% pyraclostrobin·thiophanate-methyl Suspension seed coating agent	4.1% pyraclostrobin, 36.9% thiophanate methyl	BASF, Ludwigshafen, Germany

**Table 4:** Disease incidence and disease index of the major disease affecting *Tagetes erecta* at different growth stages in Qujing, Yunnan Province.

Growing Period	Disease Name	Harmful Site	Incidence%	Disease Index
Budding stage	Brown spot	Leaves	13	5
	*Fusarium* wilt	Root, stem	6.67	3.77
Flowering period	Brown spot	Leaves, flowers, stem	18.89	10
	*Fusarium* wilt	Root, stem	8	4.44
Final flowering stage	Brown spot	Leaves, flowers, stem	71.67	50.22
	*Fusarium* wilt	Root, stem	10	5.33

**Table 5:** Regression equations, coefficients of determination (R^2^), and EC_50_ values of five fungicides against *Alternaria alternata* and FSSC.

Fungicide	*Alternaria alternata*			*Fusarium solani*		
	Regression Equation	Regression Coefficient	EC_50_ mg/L	Regression Equation	Correlation Coefficient	EC_50_ mg/L
0.3% eugenol Soluble agent	y = 0.83x + 4.51	0.939	17.518	y = 1.15x + 4.68	0.943	15.083
32% metalaxyl-M·hymexazolSeed treatment liquid	y = 0.98x + 3.43	0.956	16.961	y = 1.09x + 3.30	0.94	15.579
41% Pyraclostrobin thiophanate-methyl Suspension seed coating agent	y = 0.54x + 4.98	0.992	26.479	y = 0.49x + 4.90	0.966	27.124
30% pyraclostrobin·tebuconazol e Suspending agent	y = 0.29x + 5.43	0.704	49.604	y = 0.71x + 5.66	0.928	27.921
40% iprodione·fluazinam Suspending agent	y = 1.28x + 5.53	0.977	10.588	y = 1.09x + 7.01	0.578	12.273

## Data Availability

All relevant data are contained within the main text of this manuscript.
